# “Conflict-free communication skills” as a new academic course in the medical students’ education

**DOI:** 10.1192/j.eurpsy.2025.1540

**Published:** 2025-08-26

**Authors:** A. M. Bukinich, A. M. Konovalova

**Affiliations:** 1 Faculty of Psychology, Lomonosov Moscow State University; 2 Department of Pedagogy and Medical Psychology, Sechenov University, Moscow, Russian Federation

## Abstract

**Introduction:**

The professional activity of doctors is often associated with conflict situations, so it is important for them to obtain conflict-free communication skills during their education. These skills can be formed during a new academic course in curriculum. In this paper, we describe the prerequisites of the course “Conflict-free communication skills” on the sample of teaching pediatrics students at the Sechenov University.

**Objectives:**

To clarify the possibility and expediency integration of a new course into the curriculum of medical students.

**Methods:**

The primary method employed is bibliographic analysis.

**Results:**

The main purpose of a new course is the formation of universal, general professional and professional competencies in verbal and nonverbal communication for medical students. It is necessary for medical students to acquire the basic principles of communication psychology and the formation of conflict-free communication skills. In the process of organizing education for future medical staff, it is important to start from the basic concept of communication, not from the basic concept of conflicts. From our point of view, the concepts of effective communication and conflict-free communication are interrelated. To make this course more practical, classes should be held in the format of business games, case studies, and social-psychological training.

We have identified the following sections of the discipline:

The phenomenon of communication;

Business communication and its types;

Psychological boundaries and their violations;

Empathy in the work of a doctor;

Teamwork and collaboration.

**Conclusions:**

The integration of a new course into the curriculum of medical students will allow us to develop conflict-free communication skills and teamwork, thereby simplifying business communication in the medical field.

**Disclosure of Interest:**

None Declared

